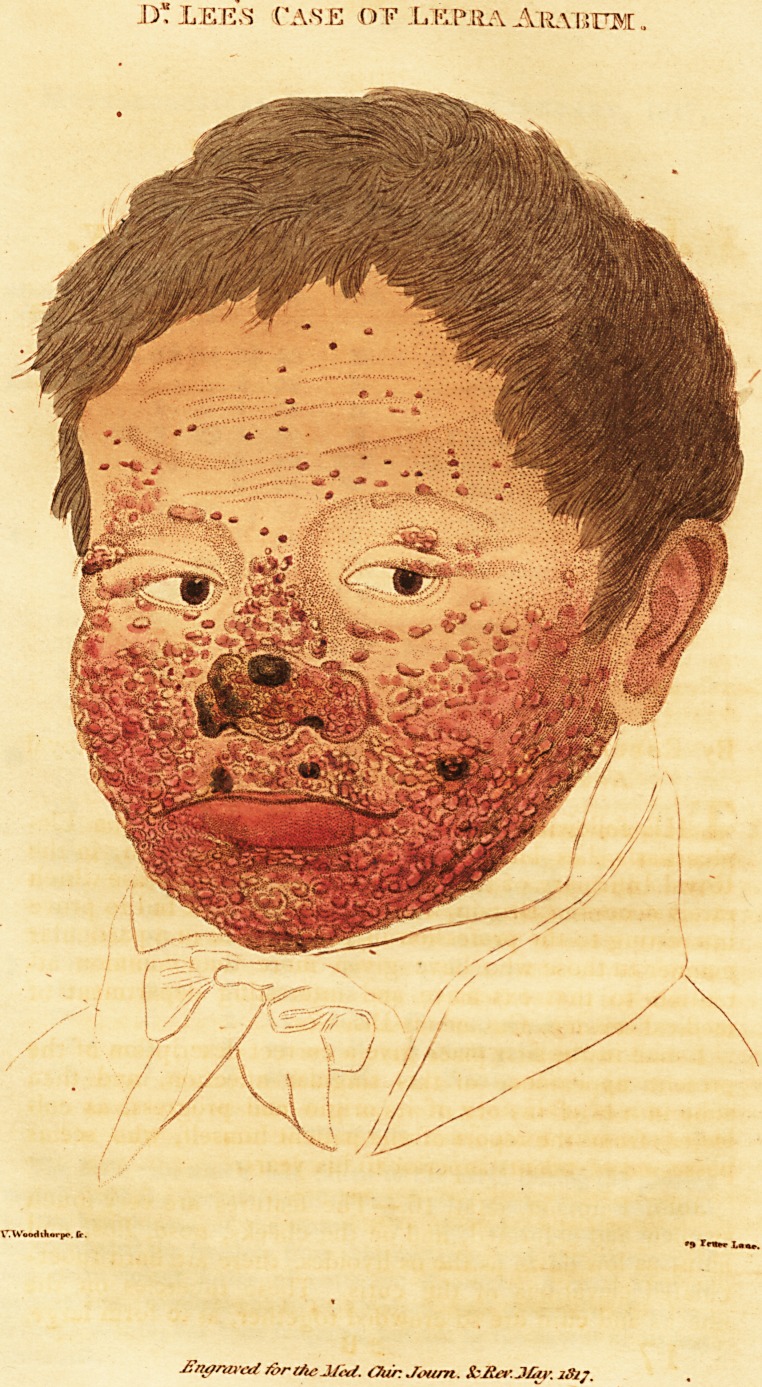# Case of *Lepra Arabum*, or True Elephantiasis

**Published:** 1817-05

**Authors:** Robert Lee

**Affiliations:** Physician's Assistant, Royal Infirmary, Edinburgh


Dr. LEE'S CASE OF LEPRA ARABUM.

V. Woodthorpe, sc.

29 Fetter Lane.

*Engraved for the Med. Chir. Journ. & Rev. May. 1817.*

THE

Medico-Chirurgical

JOURNAL AND REVIEW.

VOL. III.] MAY, 1817. [NO. 17.

### PART I.

### ORIGINAL COMMUNICATIONS.

——— quid utile ———

### *Case of* Lepra Arabum, *or True Elephantiasis.*

( With an Engraving. )

By Robert Lee, M. D. Physician's Assistant, Royal Infirmary, Edinburgh.

THE following Case of Lepra Arabum, or True Elephantiasis, has lately come under my observation, in the Royal Infirmary of this place; and as it is a disease which rarely occurs in Britain, I trust that it cannot fail to prove interesting to the profession in general, and in a particular manner to those who have given more than common attention to that extensive and interesting department of medical science, Cutaneous Diseases.

I shall in the first place give a correct description of the present appearance of this singular affection, and then subjoin a brief history of its origin and progress, as collected from the report of the patient himself, who seems possessed of talents superior to his years.

John Paterson, ætat. 16.—The features are very much swollen and deformed, and on the cheeks, nose, lips, and chin, as low down as the os hyoides, there are hard tuberculated elevations of the cutis. These tubercles on the cheeks and chin are so crowded together, as to form large, irregular, and in some places rather deep-seated lumps, and over these are placed a few smaller prominent and rounded tubercles, about the size of a garden pea; the whole presenting an appearance very similar to the rough tuberous elevations occasionally observed in potatoe-peel. These masses have an unctuous appearance and feeling, are of a dark brown or dusky colour, but those apparently more superficial are of a lighter and shining complexion, with some small florid blood-vessels ramifying on their surface. The temples retain their natural aspect, but on the forehead, which is very much wrinkled, there are situated a considerable number of flattened tubercles, varying in size from one to two lines, with general thickenings of the skin. The superciliary ridges are more than usually prominent, and the hairs are few and scattered. The upper eye-lids are thickened and tuberculated, but the cilia remain, and are of their usual appearance. The ears, particularly the lobes, helix, and anti helix, are enlarged, and occupied by a number of small tubercles. The alæ of the nose are swelled, the nostrils are preternaturally dilated, and the apex of it is entirely covered by a dark, thick, brown scab, as is a portion of the left cheek, where there is an oozing of thin fluid, which forms a light scab of a straw colour. The lips are thick and tender, discharging a thin matter in some parts, while in others scabs are forming. The voice is hoarse and weak, deglutition is painful and difficult, and, on examination, the tongue and inside of the mouth, and internal fauces and velum palati, tonsils, and uvula, are found to be occupied with numerous small white tubercles, with a considerable degree of erysipelatous inflammation of the upper surface of the tongue, roof of the mouth, and soft palate, with ulceration of the latter near the root of the uvula.

In the upper and anterior part of each thigh there is a cluster of enlarged lymphatic glands, forming a moveable, rather soft, prominent swelling; in the left, about the size of a small hen's egg, without any discolouration of the integuments, or any pain.

The thighs retain their natural form, but they are every where covered with large, irregular, discoloured, scaly patches of a dusky brown or coppery hue, with numerous small apparently subcutaneous tubercles. The legs and feet are greatly enlarged, scaly, and indurated; they are also of a dusky coppery colour, and occupied with tubercles larger and more prominent than those upon the thighs.

The superior extremities are affected in a manner similar to the thighs and legs, but the enlargement is chiefly confined to the wrists and hands; and in the bend of the right arm there is a small tumour, possessed of characters corresponding with those in the groins.

The parts of the skin thus discoloured and tuberculated are in a great measure devoid of sensibility; and piercing them with a sharp-pointed instrument, so as to produce a flow of blood, does not excite the slightest degree of pain; and at no period of the disease does it appear to have been attended with any uneasiness.

He constantly experiences a sense of constriction across the chest, with pain under the short ribs of the left side, palpitation of the heart on exertion, and slight cough with scanty expectoration. For some weeks past he has been subject to gastrodynia, cardialgia, acid eructations, with great languor, and often with faintness. The abdomen appears slightly tumid, but there is no distinct induration to be felt in any part of it. The swelling of the feet and ankles is increased towards evening, and pits on pressure. Pulse 93; tongue moist; appetite good; bowels rather costive. Urine reported to be high coloured, and deposits a copious lateritious sediment.

He was born in the island of St. Christopher's; his father is a native of the West of Scotland, his mother a white woman, likewise born in that island, and remarkably healthy. From infancy he has been particularly delicate and feeble, so that he has been unable to partake in the amusements of boys of the same age; has been often subject to pain in his back, to loss of appetite, and for a year preceding the present ailment, to weakness of vision. He sailed from the West Indies for Scotland on the 27th of June, 1813, and landed in this country on the 12th of August following.

About six days subsequent to his arrival, both legs began to swell, and the integuments of the right became hard and insensible. At the same time he suffered much from pain in the left side, immediately below the false ribs. Five weeks afterwards, some small pimples, accompanied with itching and tingling, arose about his wrists, which he at first supposed to be the prickly heat (*Lichen Tropicus*) of the West Indies, to which he had been exceedingly liable while in that climate.

The integuments of the superior extremities, particularly of the wrists and fore-arms, now also began to grow thickened and discoloured, while the affection of his legs continued to increase; and above the right ankle, where the cutis had, as he describes it, the feel of leather, an oozing of a fluid took place, which speedily concreted into thin yellowish scabs. In about nine months, the symptoms still increasing, the integuments of his face became affected, a hard tumour having appeared upon the middle of his nose, about the size of a black currant, of a shining red colour, and situated apparently under the skin.

In June, 1815, he had a severe attack of measles, and during the two weeks of his confinement, the tubercle on his nose almost entirely disappeared. He had not, however, completely recovered, before this tubercle again increased to its former size, and another appeared below the left eye, accompanied with considerable heat, redness, and tenderness of the whole face. In the course of three months, both tubercles having now become elevated, clear, shining, and soft, they opened spontaneously, and discharged a quantity of bloody ichor.

He continued in this situation without any material change in the symptoms until February 1816, when the skin of the face began to swell and thicken, and numerous flattened tubercles to arise on the cheeks, ears, lips, and chin. From small openings in these tubercles, now gradually increasing in number and size, a copious discharge occurred, which formed thick dark brown scabs over the whole of the face, except the forehead: these scabs soon fell off, and a fresh discharge took place, succeeded also by a similar scabby incrustation.

During last summer, he thinks the discharge from his feet and legs was much increased, and wherever the integuments had the leathery feel, numerous chaps or fissures appeared, which were very itchy, and irritated by the heat of the fire. The general thickening of the skin on his thighs was nearly synchronous with the affection of his ankles. During the last summer also, the discharge from his face was materially increased.

About September and October, however, there was a considerable improvement of all the symptoms. In this apparent amendment, he continued till the commencement of the winter; then the general cutaneous affection became rather worse than before. The discharge from the tubercles on his face, and under the chin, was so much increased as to stain the pillow; an increase of pain was always felt during frosty weather. He is uncertain when his mouth and throat first became covered with eruption, but recollects to have felt hoarseness and pain on deglutition ever since the disease appeared on his face. His general health, until of late, has been good; but six weeks before his admission into the hospital, he observed his urine discoloured and thick, and shortly afterwards felt severe general pains, particularly fixed in the epigastric region, and great debility. Previous to his admission, he has been using, by medical advice, the warm sea-water bath, which had no other effect than that of bringing off the scabs from the subjacent small tubercles. These soon produced a fresh crop of dark brown scabs. He has also undergone two courses of mercury, but without any benefit.

He was admitted into the hospital on the 10th instant, and he is at present using mild diaphoretics and calomel, and antimony in small doses, in the form of Plummer's pill, with a moderate allowance of wine and nourishing diet, without any amelioration of the complaint.

Since the time of his admission into the hospital, the discharge from his face has been abundant, and it is now covered partially with crusts. His health has been declining rapidly, and for some days he has been much afflicted with the pain in his left side, with frequent attacks of hard, dry cough; while he perspires profusely under night, and the œdema of the lower extremities towards evening ascends as high as the knees. His pulse has generally been betwixt 90 and 100, and his urine is almost white like milk, and deposits a copious sediment of the same colour.

The organs of generation appear to be nearly in the same condition as they are usually observed to be in boys before the age of puberty, with the exception of the scrotum, which, though nearly empty, is considerably larger. The testicles are small and soft, and are situated high up, near the external abdominal aperture. He reports that they have diminished nearly one half in size, and are still becoming smaller. There are no hairs on the pubis or chin. The prepuce is covered both on the inner and outer surface with tubercles, and is so much contracted that the glans cannot be completely uncovered.

The disease, in this case, does not appear to be infectious, as this boy has slept in the same bed with his brother during the whole period that he has been affected with the complaint, and in him none of the symptoms have occured.

ROBERT LEE, M. D.

Edinburgh, March 24, 1817.

## Figures and Tables

**Figure f1:**